# Within- and Among-Population Variation in Chytridiomycosis-Induced Mortality in the Toad *Alytes obstetricans*

**DOI:** 10.1371/journal.pone.0010927

**Published:** 2010-06-02

**Authors:** Ursina Tobler, Benedikt R. Schmidt

**Affiliations:** 1 Institute of Evolutionary Biology and Environmental Sciences, University of Zurich, Zurich, Switzerland; 2 KARCH, Neuchâtel, Switzerland; University of Sao Paulo, Brazil

## Abstract

**Background:**

Chytridiomycosis is a fungal disease linked to local and global extinctions of amphibians. Susceptibility to chytridiomycosis varies greatly between amphibian species, but little is known about between- and within-population variability. However, this kind of variability is the basis for the evolution of tolerance and resistance evolution to disease.

**Methodology/Principal Findings:**

In a common garden experiment, we measured mortality after metamorphosis of *Alytes obstetricans* naturally infected with *Batrachochytrium dendrobatidis*. Mortality rates differed significantly among populations and ranged from 27 to 90%. Within populations, mortality strongly depended on mass at and time through metamorphosis.

**Conclusions/Significance:**

Although we cannot rule out that the differences observed resulted from differences in skin microbiota, different pathogen strains or environmental effects experienced by the host or the pathogen prior to the start of the experiment, we argue that genetic differences between populations are a likely source of at least part of this variation. To our knowledge, this is the first study showing differences in survival between and within populations under constant laboratory conditions. Assuming that some of this intraspecific variation has a genetic basis, this may suggest that there is the potential for the evolution of resistance or tolerance, which might allow population persistence.

## Introduction

Emerging infectious diseases can represent an important threat to biodiversity and wildlife [1]. Although extinction by disease is a rare event and unlikely in most systems, certain conditions can increase the risk of local or even global extinction by disease: a sympatric host that acts as a reservoir for the pathogen, disease transmission that is frequency-dependent, or disease that drives local populations to such low densities that stochastic factors become important [2].

Chytridiomycosis, a disease caused by the chytrid fungus *Batrachochytrium dendrobatidis* (*Bd* hereafter), is a disease of amphibians that may cause extinctions because it fulfils all three conditions: It infects a variety of hosts with different susceptibilities, some of them acting as reservoirs [3], transmission is frequency-dependent at low densities [4], and amphibians often occur in small populations that are prone to stochastic effects [5]. Accordingly, chytridiomycosis has been linked to local and global extinctions of amphibians in Central America, Australia and Europe since its discovery in 1998 [6].

Susceptibility to *Bd* and chytridiomycosis varies greatly among amphibian species [7] and much research has been devoted to the identification of the causes of this variability (e.g. [8], [9], [10]). This line of research helps to identify which species are at risk from *Bd* [11]. While some species seem to have disappeared completely following *Bd* invasion [12], populations of other species have persisted [13], [14], [15], suggesting that there is variation in susceptibility to disease within species. Here we focus on within-species variation in susceptibility to chytridiomycosis because this kind of variability is the raw material for the possible evolution of resistance or tolerance [16] to *Bd*, and hence population persistence [17], [18], [19]. As no methods are available yet to treat amphibian populations in the field against *Bd*, susceptible species may persist only where conditions are not favourable for *Bd* or for disease outbreaks [20], [21], or when they can evolve an evolutionary response to the threat imposed by the emergence of chytridiomycosis. The latter may include evolutionary change in defensive skin peptides. Nevertheless, only few studies have addressed within-species variation in susceptibility to this disease and none has looked at the genetic basis of this variation [7], [22], [23].

One of the species that is known to be highly susceptible to chytridiomycosis is the common midwife toad, *Alytes obstetricans*. The species is widespread across Europe and has suffered substantial declines throughout its range [24]. It was the first species to decline in Europe as a result of mass die-offs after the emergence of *Bd* [25]. In Spain, mortality almost exclusively affected recently metamorphosed individuals of this species, as the adults are strongly terrestrial and have a low risk of infection. Chytridiomycosis-induced mortality is strongly associated with cold and moist conditions at the time of metamorphosis [20], [21]. Midwife toads have also suffered strong declines in Switzerland, where more than 50% of the populations went extinct over the last quarter century [26]. *Bd* is present and widespread in Switzerland ([27], U. Tobler & B. R. Schmidt, unpublished data).Yet, in the field, no mass mortality has been observed. Only four dead metamorphs were found in the field so far, all of which exhibited high zoospore loads as determined by rt-PCR (B. R. Schmidt & U. Tobler, unpublished data).

The aim of our study was to quantify the impact of *Bd* and variation thereof on midwife toad populations by measuring post-metamorphic survival of infected individuals in a common garden laboratory experiment. We demonstrate that *Bd* is linked to mortality under laboratory conditions and that mortality varies greatly among populations. We also identify correlates of mortality among individuals within populations.

## Materials and Methods

### Study sites

We collected one-year old tadpoles of *Alytes obstetricans* shortly after hibernation in 2008 from three sites in Switzerland, two of which were located in canton Baselland and one in canton St. Gallen. Standard hygiene protocols were followed during field work to avoid the spread of *Bd* and other pathogens [28]. The sites were chosen because 1) we knew they sustained large tadpole numbers and 2) they were positive for *Bd* [28]. The first site in Baselland (7.783306°E, 47.459667°N, 410 masl, hereafter referred to as BLI) is located in a former quarry and consists of three main water bodies, in only two of which tadpoles were caught. The larger pond is about 16 m^2^ in size, 0.8 m deep and is densely vegetated; the smaller pond is 8 m^2^ in size, 0.5 m in depth, and has very little vegetation but has reed along the edges. The second site in Baselland (7.801185°E, 47.436677°N, 480 masl, hereafter referred to as BLZ) is a forest pond approximately 300 m^2^ in size and 2.5 m in depth. The site in St. Gallen (9.533416°E, 47.381742°N, 540 masl, hereafter referred to as SGA) is a garden at the south-eastern distribution border of the species. The population is larger than 20 calling males, and three small garden ponds (2–5 m^2^ in size, 0.2–0.5 m in depth) serve as breeding sites.

### Laboratory experiment: treatments

*Bd* only infects keratinised skin of amphibians [29] and infection in tadpoles is restricted to the mouthparts, which does not cause disease. However, during metamorphosis, the skin becomes keratinised, and the pathogen can then spread over the whole body and cause hyperkeratosis and osmotic imbalance, leading to death [30], [31]. Recently metamorphosed individuals are most susceptible to disease, supposedly due to a downregulation of immune defences during metamorphosis [32]. Thus, we measured differences in post-metamorphosis survival of *Bd*-positive and -negative *Alytes* tadpoles from the three populations. Because all tadpoles were tested for *Bd* infection with rt-PCR [33] and confirmed positive initially, we assigned them to three different treatments in order to obtain one *Bd*-negative control: 1) The “Itraconazole treatment” group served as a *Bd*-free control. Individuals were treated with Itraconazole (1 mg/L; Sporanox, Janssen-Cilag) for 5 minutes per day over the course of seven days to clear the infection. We followed the protocol developed by Garner et al. [34] which had been successfully used for the treatment of *Alytes muletensis* tadpoles against *Bd*; unlike Garner et al. [34], we observed no depigmentation. The tadpoles were returned to the same container after treatment, which had been rinsed with boiling water during the Itraconazole bath. 2) Because the tadpoles were exposed to low water levels and frequent capture during the Itraconazole treatment, we designed the “infected handled treatment” as a control for stress during handling. The tadpoles were treated in exactly the same way as the Itraconazole treatment, but instead of an Itraconazole solution, tap water was used. 3) The “infected unhandled” group was not handled apart from the regular water change and feeding and was left infected throughout the experiment.

To test for differences in survival among populations, we assigned equal numbers of tadpoles from the three populations to the three treatments. We balanced body mass among treatments within populations.

### Laboratory experiment: procedures

We caught 143 tadpoles (50 from BLI, 43 from BLZ and 50 from SGA) in April 2008 and brought them to the laboratory, where they were placed in individual one-litre plastic containers filled with tap water and a single dried beech tree (*Fagus* sp.) leaf. The laboratory was equipped with full spectrum sunlight lamps, and we used a daylength of 16 hours and kept the room at 19–21°C. Throughout the course of the experiment, we changed the water in the containers twice a week and added food containing *Spirulina* algae (Tetra Pleco Wafer, Tetra Germany, Spectrum Brands Inc.) *ad libitum* three times a week. In the week following capture, the tadpoles were weighed to the nearest 0.01 g (Scaletec Instruments, Heiligenstadt, Germany), measured from the snout to the beginning of the tail muscle to the nearest 0.1 mm, and swabbed over the mouth parts with a sterile cotton swab (Copan Italia S.p.A., Brescia, Italy). Further swabs were taken when metamorphosis was finished (Gosner stage 46, [35]) and (3) upon death or 30 days after stage 42. Size measurements were repeated three times during the experiment: (1) when the larvae entered metamorphosis (Gosner stage 42), (2) when metamorphosis was finished (Gosner stage 46) and (3) upon death or 30 days after stage 42. We used separate pairs of gloves for handling and separate plastic beakers for weighing each tadpole. When the tadpoles entered metamorphosis (Gosner stage 42), the containers were drained of most of the water and tilted, so that both land and water were available to the toadlets. We put a moist paper towel at the bottom of each container. When the metamorphosis was complete (Gosner stage 46), we fed the toadlets crickets of adequate size *ad libitum* three times a week. For each individual, the experiment was ended 30 days after stage 42 or upon death.

### rt-PCR

We analysed the swabs for the presence of *Bd* with *Bd*-specific primers in rt-PCR following the protocol by Boyle *et al*. [33] with slight modifications: samples were run in duplicates and when the two PCR-wells retuned inconsistent results, the analysis was repeated. Because the extraction reagent is inhibitory to the PCR reaction, samples were diluted 1∶10 prior to rt-PCR analysis. As in Boyle et al. [33], we report untransformed zoospore equivalents. Reactions yielding 0.1 genomic equivalents (GE; untransformed value) or above were considered *Bd*-positive. This threshold was used to classify individuals as either “infected” or “not infected”. For the statistical analyses, however, we used zoospore loads rather than the categories “infected” or “not infected”.

### Statistical analyses

We tested for differences in body length among the different populations at three stages during development: at the beginning of the experiment, at stage 42 (beginning of metamorphosis) and at stage 46 (end of metamorphosis) using separate ANOVA, and we tested for correlations between body mass and length at stage 42, 46 and the end of the experiment. Because mass and length were strongly correlated, we only used body mass for subsequent analyses.

To analyse survival data we used the Cox proportional hazard model. This model assumes an underlying hazard function describing how hazard changes over time, and fits effect parameters using Cox's likelihood. Individual survival times are censored, which means that individuals may die after the end of the study period. We tested for overall differences in mortality between treatments and populations using daily counts on the number of survivors until 30 days after beginning of metamorphosis (Gosner stage 42). To test for the effects of individual condition and environmental effects on hazard risk, we did a Cox proportional hazard model with the two infected treatments only. The variables on individual condition included were body mass at the beginning of the experiment, at stage 42, 46 and at the end or death, and time through metamorphosis; the variables for environmental effects were zoospore loads at the beginning, at stage 46, at the end and time to metamorphosis. We also included interactions between zoospore loads and population.

Because individuals that survived until the end of the experiment had more time to gain mass, we tested the correlation between body mass and time since metamorphosis with a Pearson correlation test. All tests were performed in R, version 2.8.1 [36].

## Results

**Figure 1:**
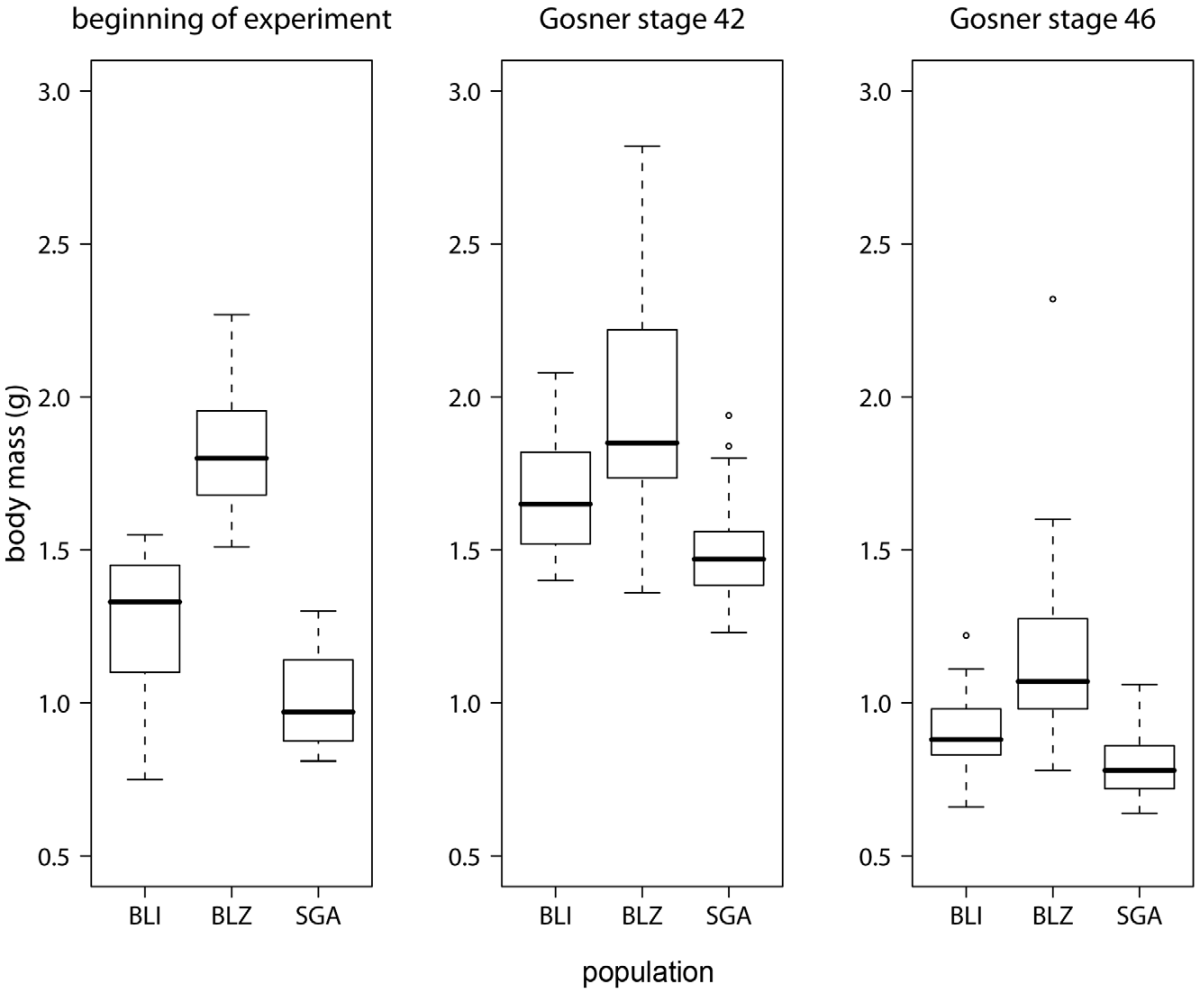
Differences in body mass between populations. Boxplots showing body mass of the different populations at the beginning of the experiment, at the start of metamorphosis (Gosner stage 42) and at the end of metamorphosis (Gosner stage 46). The black line represents the median, the box represents the interquartile range containing 50% of the values, and whiskers mark the 1.5 fold interquartile range. Outliers are marked with circles. Grey boxes = survivors, white boxes = non-survivors.

Populations differed significantly in body mass at the beginning the experiment (ANOVA for mass at beginning of the experiment and at stage 42: both p<0.001) but not at metamorphosis (ANOVA at stage 46: p = 0.144; Figure 1). Body length and mass were strongly correlated at all stages (Pearson correlation for beginning, stage 42, stage 46 and end of experiment; r_beginning_ = 0.83, r_stage___42_ = 0.76, r_stage_46_ = 0.30, r_end_ = 0.90; all p<0.001).

All 143 overwintered tadpoles were infected with *Bd* at the time of capture. When they completed metamorphosis (Gosner stage 46), all Itraconazole-treated tadpoles were *Bd*-negative while all infected unhandled or infected handled tadpoles were still infected. Eight tadpoles died during the course of the experiment before undergoing metamorphosis: Two tadpoles from BLI died before treatment assignment within a week after capture, and another tadpole from BLI died in the unhandled group. Two tadpoles from BLZ died in the Itraconazole group, and from SGA one tadpole died in the Itraconazole treatment and two from the infected handled treatment, the last five all within 1.5 weeks after treatment. Because chytridiomycosis usually does not cause mortality during the larval stage and most deaths were probably caused by transportation or handling stress, these 8 tadpoles were excluded from the analyses. The remaining 135 tadpoles reached Gosner stage 42 (beginning of metamorphosis) 25 to 129 days after 25 April, when the first swab was collected (and 32–136 days after capture). From stage 42 on, it took them seven to 18 days (mean±SD: 12±2) to fully resorb the tail.

All Itraconazole-treated toadlets survived until the end of the experiment; mortality only occurred in infected toadlets. Across all populations, only 34.1% (31 out of 90) of infected (handled and unhandled) metamorphs survived until 30 days after stage 42; those that died survived on average for 8.6±6.5 days post-metamorphosis only.

Infected animals that died showed disease symptoms typical of chytridiomycosis shortly before death [37]. Diseased individuals stopped feeding approximately one day prior to death but did not show any other symptoms until less than a day before they died. Only within a few hours to death they would become lethargic and loose their righting reflex, at the same time they started to shed skin heavily when touched. Although we did not investigate dead individuals histologically to confirm that chytridiomycosis was the cause of death, disease symptoms and severity of infection as quantified by rt-PCR shortly before or after death, respectively, suggest that chytridiomycosis was the cause of death.

**Figure 2:**
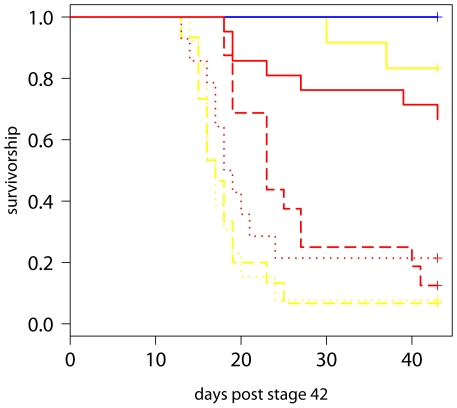
Survival curves for treatments and populations. Cox regression on survival depending on treatment and population. The blue line represents the Itraconazole treatment for all populations. Yellow = infected handled, red = infected unhandled. Continuous line = population BLI, dashed line = population BLZ, dotted line = population SGA.

The Cox proportional hazard test revealed a significant effect of treatment (p<0.001) and population (p<0.001) on survival (Figure 2). Survival differed between infected and uninfected individuals, but not between the infected unhandled and the infected handled group (unhandled: 33.5±29.1%, handled: 32.6±43.9%). Population BLI differed from the two other populations in survival among infected individuals (i.e. confidence intervals did not overlap; BLI: 75±2.0% (SE), BLZ: 14.6±1.9%, SGA: 9.6±0.7%), but there was no difference in survival between SGA and BLZ. Because the handled and unhandled group did not differ in survival, they were pooled for subsequent analyses.

**Figure 3:**
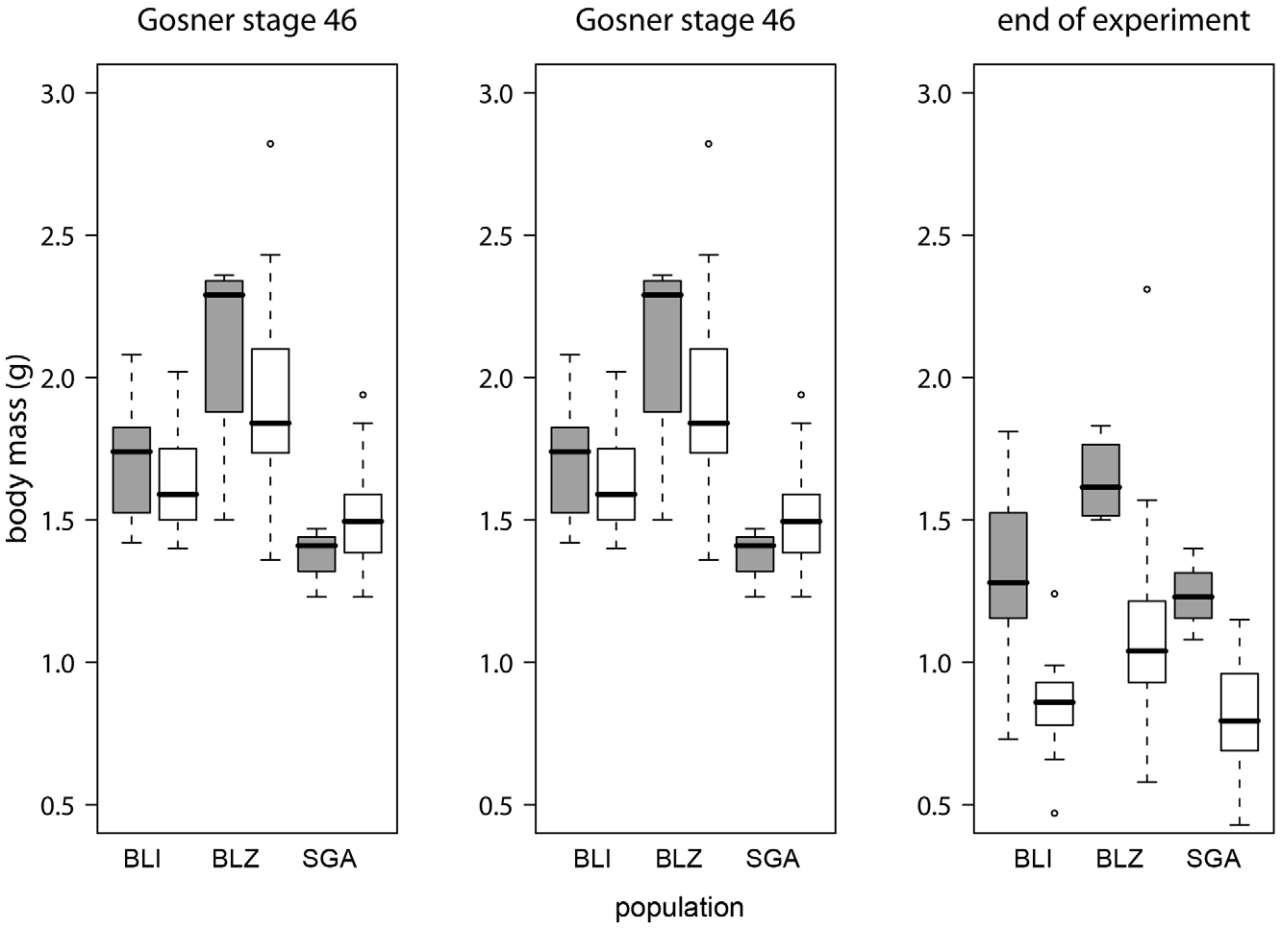
Body mass in surviving and non-surviving individuals. Boxplots showing body mass of infected individuals at the beginning of metamorphosis (Gosner stage 42), at the end of metamorphosis (Gosner stage 46) and at the end of the experiment or death. Grey boxes = survivors, white boxes = non-survivors. The black line represents the median, the box represents the interquartile range containing 50% of the values, and whiskers mark the 1.5 fold interquartile range. Outliers are marked with circles. Grey boxes = survivors, white boxes = non-survivors.

**Figure 4:**
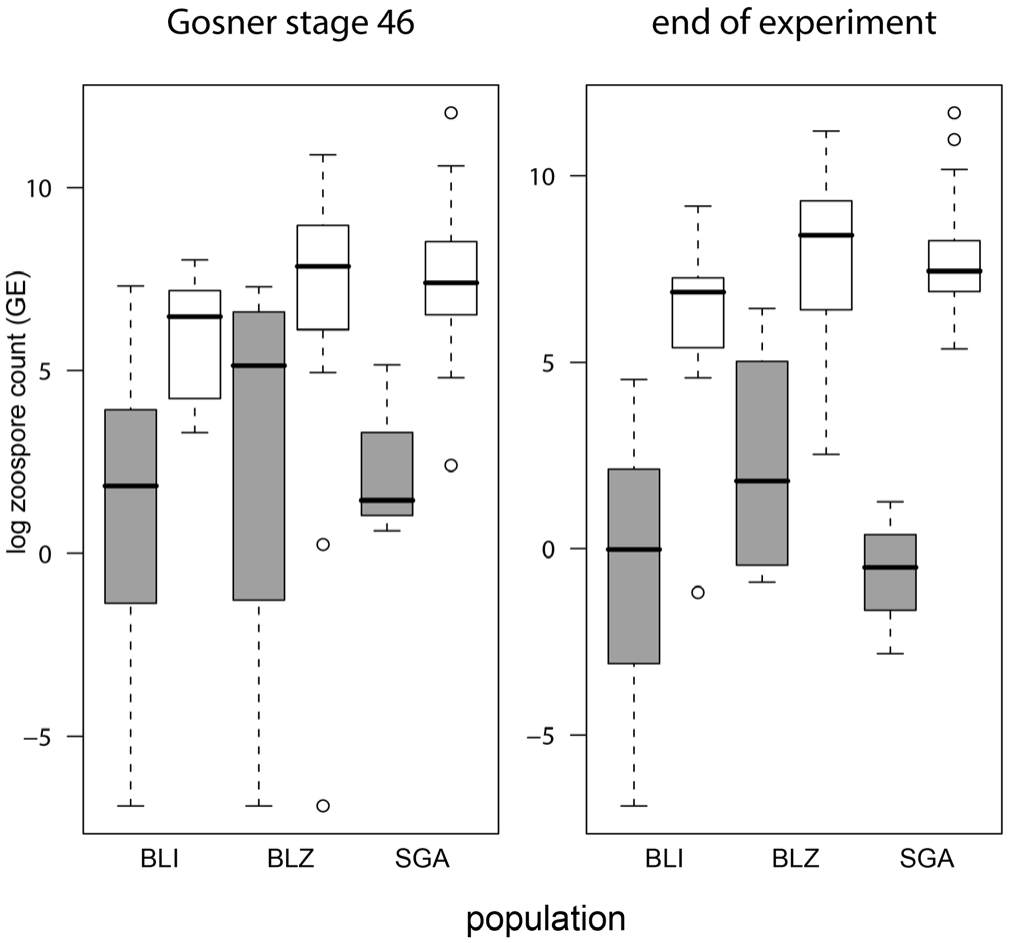
Infection loads of surviving and non-surviving individuals. Infection load, measured in genomic equivalents from rt-PCR reactions and logarithmically transformed, in individuals from the infected treatments at the end of experiment. The black line represents the median, the box represents the interquartile range containing 50% of the values, and whiskers mark the 1.5 fold interquartile range. Outliers are marked with circles. Grey boxes = survivors, white boxes = non-survivors.

In the infected treatment groups, heavier individuals survived better (Cox proportional hazard test, Figure 3, Table 1); body mass at the end of the experiment strongly correlated with life span since stage 46 (Pearson correlation, p<0.001). Survivors completed metamorphosis slightly slower than individuals that died later on (survivors: 12.9±2.8 (SD) days, non-survivors: 12.3±2.6 days, Table 1). Although infection load at any stage did not affect individual hazard risk (Cox proportional hazard test, Table 1), non-survivors had on average higher zoospore loads (9701 GE±21726.2) than survivors (28.2 GE±113.2; Figure 4) at death or at the end of the experiment, respectively. There was an interaction between zoospore load and population (Table 1, Figure 4). Three individuals out of 31 survivors had cleared infection below the detection threshold.

**Table 1:** Summary of the results of the Cox regression survival analysis of the two treatments with infected individuals.

	Mean±SD		Test statistic				
Source of variation	Survivors	Non-survivors	coef	exp(coef)	se(coef)	z	p
Population	-	-	4.37	244.10	1.05	4.03	0.000
Mass beginning	1.31±0.33	1.37±0.41	−5.18	0.01	1.83	−2.83	0.005
Mass stage 42 (g)	1.72±0.29	1.69±0.31	2.71	15.00	1.33	2.04	0.041
Mass stage 46 (g)	0.98±0.30	0.96±0.22	−1.62	0.20	1.50	−1.08	0.280
Mass end (g)	1.36±0.28	0.93±0.29	−1.06	0.35	0.79	−1.35	0.180
Time to stage 42 (days)	64.84±31.59	52.85±13.13	0.00	1.00	0.00	−0.04	0.970
Time from stage 42 to 46 (days)	12.87±2.81	12.28±2.58	0.00	1.00	0.00	3.41	0.001
Zoospore load beginning (GE)	661.20±2081.79	677.79±1008.66	0.00	1.00	0.00	2.49	0.013
Zoospore load stage 46 (GE)	155.96±376.63	8496.52±23779.21	−0.04	0.96	0.02	−2.22	0.027
Zoospore load end (GE)	28.19±113.21	9700.93±21726.15	0.00	1.00	0.07	−0.01	0.990
Population*zoospore load stage 46	-	-	0.00	1.00	0.00	−3.36	0.001
Population*zoospore load end	-	-	0.00	1.00	0.00	−2.44	0.016

Mean values and Cox proportional hazard test results analysing the impact of body mass at the beginning, at Gosner stages 42, 46 and at the end of experiment, time to and trough metamorphosis and zoospore load (genomic equivalents, GE) at the beginning, stage 46 and the end of the experiment.

## Discussion

Here we show that under laboratory conditions, mortality in *Bd*-infected metamorphs was population-specific and varied from 27% to 90%. Our experiment thus confirms that *Bd* infection was linked to substantial mortality in *Alytes* toadlets from populations where no mass mortality events caused by chytridiomycosis have been reported even though *Bd* is widespread ([27], U. Tobler and B. R. Schmidt, unpublished data). If the observed mortality is representative of *Bd*-induced mortality in the field, such high levels of mortality may lead to population declines [38], [39].

How could such populations persist? Sensitivity analyses of amphibian life histories suggest that post-metamorphic juvenile survival appears to determine the fate of the populations. High levels of mortality may lead to population declines [38], [39]. In contrast, Briggs et al. [13] modelled the effects of *Bd* on population dynamics and predicted population persistence if some infected individuals survive. Because no data on population trends are available for the populations that we studied, we cannot tell whether they currently undergo declines. To predict the fate of our study populations, more data would be necessary. For example, it is unknown (1) for how long *Bd* has been present in these populations, (2) how long after the onset of chytridiomycosis the adult populations are expected to decline, (3) whether and how strongly mortality rates differ between years and (4) whether density dependence in the adult stage could dampen the effects of *Bd*-associated mortality immediately after metamorphosis.

To our knowledge, this is the first study to show differences in the survival of *Bd*-infected individuals both within and between populations in the laboratory. We do not know why individuals and populations differed in susceptibility. Some variation may be attributable to variation among individuals in growth (i.e., mass at metamorphosis) and development (i.e., duration of metamorphosis; [40], Table 1 and Figure 3), although the large difference in body mass at the end of the experiment mostly resulted from the longer lifespan of survivors (Figure 4). Survivors and non-survivors differed in the duration of metamorphosis (Table 1). Duration of metamorphosis may be an important determinant of how quickly the immune system recovers after metamorphosis [32]. A better understanding of how duration of metamorphosis affects *Bd*-associated mortality would be a worthwhile topic for further study. Variation in mass and survival may have been caused by differential environmental conditions early in the larval stage, by variation in the tadpole immune system (i.e. genetic variation at disease resistance or tolerance loci, antimicrobial peptides and symbiotic bacteria), because of differences between *Bd* strains from the tadpoles' sites of origin or because of infection of single vs. multiple *Bd* strains.

We do not expect that environmental conditions experienced early in life affected the outcome of the experiment. The tadpoles were captured after hibernation. We kept them in the laboratory under common garden conditions for 4 to 18 weeks before they started metamorphosis. Because effects of previously experienced environmental conditions often fade out quickly [41] and because the common conditions in the laboratory experiment would minimise environmental variation, the effects of previously experienced environmental conditions in the ponds of origin should be minimal.

The immunocompetence is likely to have varied among individuals and populations. While we know little about the genetic basis of the immune system in amphibians [42], we know that antimicrobial peptides and symbiotic bacteria that are active against *Bd* may vary both among individuals and populations [43], [44]. Strains of *Bd* are known to differ in how much mortality they inflict on amphibians [45] and multiple strains of *Bd* may occur within the same locality [21], [46]. Infection with multiple strains might affect virulence [47], [48]. We did not test whether tadpoles in our experiment had the same *Bd* strain(s), antimicrobial peptides, or bacteria. We know, however, that the bacterial communities differ between the populations BLI and BLZ (L. Davis, personal communication).Whether and how the bacterial communities found at each site differ in their *Bd*-inhibitory effects is the subject of current research (L. Davis, personal communication). For logistic reasons during experimental work, the same equipment was used for water changes for all populations within one treatment, and thus bacterial communities and pathogen strains may have been homogenised. Nevertheless, skin microbiota may account for some of the variation in survival that we observed [22]. We suggest that some of the variation in mortality among populations that we observed in the common garden experiment is due to genetic differences among populations. This is the usual interpretation of among-population variation in common garden experiments. Two lines of evidence support this suggestion. First, we know that the host populations are genetically differentiated (pairwise F_ST_ based on 12 microsatellites ≥0.19, U. Tobler & B. R. Schmidt, unpublished data). Second, individual differences in growth (mass at metamorphosis) did affect survival; among-population variation in life history traits can have a genetic basis [49]. Considering this, genetic differences among populations may at least partially explain the observed differences in mortality, as it is often the case in host-parasite associations [50], [51].

A number of experimental studies reported that many *Bd*-infected amphibians can survive or even clear infection (see electronic appendix to reference [7]). For example, Fisher et al. [45] reported that host survival varied in a dose-dependent manner among *Bd* strains. In most cases, a substantial proportion of hosts survived (as in our experiment; Figure 1). Nevertheless, *Bd* imposes strong selection on amphibian hosts. If variation in susceptibility to *Bd* has a genetic basis, we would expect to see genetic changes in the host population and ultimately the evolution of resistance or tolerance to *Bd* [16], [52], [53]. Such pathogen-mediated selection is known to occur in amphibians: Tennessen and Blouin [44] and Teacher et al. [54] showed that there is natural selection on the genetic diversity of antimicrobial peptides and MHC alleles, respectively. One interpretation of among-population variation in *Bd*-associated mortality reported in Figure 1 is that the populations we studied may already differ in their degree of tolerance or resistance to *Bd*.

While strategies to manage *Bd* in the wild are still being developed [3], [7], we suggest that enhancing an evolutionary response of amphibians to *Bd*, may be a worthwhile conservation strategy to mitigate the effects of the disease [55]. The model by Briggs et al. [13] suggests that amphibian populations can persist or even recover if some individuals loose the infection. Such a “waiting for resistance or tolerance to evolve” strategy may be risky, however, as some hosts may fail to evolve adaptations to novel pathogens [18]. First, rapid disease emergence may cause amphibian populations to go extinct in many places before resistance or tolerance allowing for population persistence can evolve [18], [56], [57]. Second, *Bd* is likely to evolve counteradaptations to host resistance [45]. Yet, if *Bd* and amphibian hosts would enter a coevolutionary process, then extinction–as commonly observed in areas where *Bd* emerged–may become a less likely outcome.

In summary, our experiment demonstrates that within a species, mortality can greatly differ both at the population and individual level, and that these different mortalities are not necessarily due to climatic variation at the time of metamorphosis because in this case they were observed under stable laboratory conditions. The results also show that *Bd*-associated mortality can be substantial in an area where *Bd* is widespread ([27], [28], U. Tobler and B. R. Schmidt, unpublished data) but where no mass mortalities or *Bd*-associated population declines have been reported [26]. Nevertheless, the high mortality rates we observed are likely to affect populations and suggest that *Bd* may be a cryptic driver of amphibian population dynamics. The mechanisms how amphibian populations can cope with additional mortality due to chytridiomycosis are unknown. We argue that in many situations, global or local extinction will only occur if aided by other threats such as habitat degradation, demographic stochasticity or unusual weather conditions. We suggest that conservation measures should prioritise populations that have high resistance or tolerance against chytridiomycosis to prevent the loss of these populations by other threats. In the long term it is desirable to determine what factors are involved in population level disease resistance or tolerance in order to allow the transfer of resistance mechanisms, such as resistant genotypes or symbiotic skin microbiota, into non-resistant populations. This may enable the management of amphibian populations, or their habitat, to increase survival rates and thus allow long-term population survival in the presence of novel disease threats.
