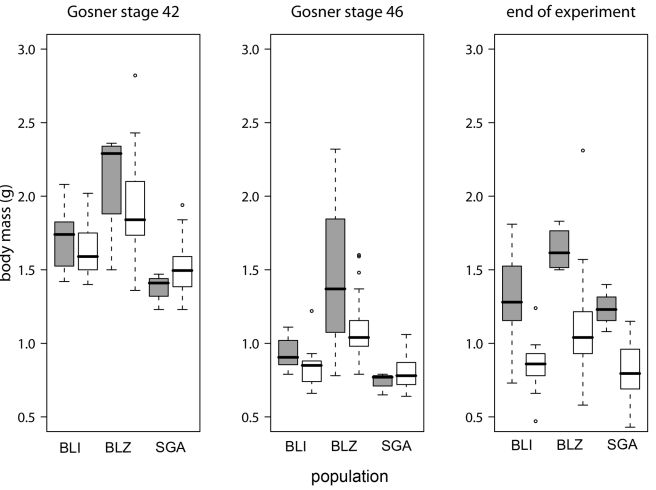# Correction: Within- and Among-Population Variation in Chytridiomycosis-Induced Mortality in the Toad *Alytes obstetricans*

**DOI:** 10.1371/annotation/3708df43-0eb2-44bf-8589-a153c199d35d

**Published:** 2010-07-09

**Authors:** Ursina Tobler, Benedikt R. Schmidt

Figure 3 contains an error. Please view the correct figure here: 

**Figure:**